# Alterations of Total Serum Immunoglobulin Concentrations in Pemphigus and Pemphigoid: Selected IgG2 Deficiency in Bullous Pemphigoid

**DOI:** 10.3389/fmed.2020.00472

**Published:** 2020-09-02

**Authors:** Stanislav Khil'chenko, Katharina Boch, Nina van Beek, Artem Vorobyev, Detlef Zillikens, Enno Schmidt, Ralf J. Ludwig

**Affiliations:** ^1^Lübeck Institute of Experimental Dermatology, University of Lübeck, Lübeck, Germany; ^2^Center for Research on Inflammation of the Skin, University of Lübeck, Lübeck, Germany; ^3^Department of Dermatology, University of Lübeck, Lübeck, Germany

**Keywords:** pemphigus, pemphigoid, immunoglobulin, IgG, IgM, IgA, ELISA, primary antibody deficiency

## Abstract

Pemphigus and pemphigoid diseases are organ-specific autoimmune diseases of the skin and/or mucous membranes, which are caused by autoantibodies targeting structural proteins of the skin. In other autoimmune diseases, a high prevalence of primary antibody deficiencies was noted. Conversely, a high prevalence of autoimmune diseases is reported in patients with primary antibody deficiencies. With the exception of one study, pointing toward a decrease of IgG in pemphigus patients, with a relative enrichment of IgG4, serum immunoglobulin (Ig) concentrations had not been studied in pemphigus and pemphigoid. Hence, we here aimed to investigate serum concentrations of IgM, IgA, IgG, and IgG1–4 in pemphigus and pemphigoid patients, as well as in healthy controls. Serum Ig concentrations were determined by ELISA in 105 healthy controls, 100 pemphigus vulgaris (PV), 100 pemphigus foliaceus, 99 bullous pemphigoid (BP), and 55 linear IgA bullous dermatosis (LAD) patients. In healthy controls, age had a significant impact on Ig serum concentrations: In controls at ages of 69 years or older, IgM and IgG were decreased, while all other Ig, except IgA and IgG4, were increased. When stratified by sex, lower IgM concentrations were observed in males. When corrected for age and/or sex, and compared to controls, an increase in serum IgA was noted in LAD. In almost all patient cohorts, an increase in IgG1 and IgG4 was observed, while a decrease in IgG2 or IgG3 was seen in BP or PV patients. This points toward a possible association of BP with IgG2 deficiency and warrants evaluation of IgG2 in BP patients prior to immunosuppressive therapy.

## Introduction

Pemphigus and pemphigoid diseases are prototypical organ-specific skin autoimmune diseases, characterized and caused by autoantibodies targeting defined structural proteins expressed in the skin and/or mucous membranes (1–3). Depending on the clinical presentation and the specificity of the autoantibodies, distinct pemphigus, and pemphigoid diseases are diagnosed (4). Overall, pemphigus and pemphigoid are rare diseases but have a significant impact on patients' morbidity and mortality (5) with a high and, so far, unmet medical need (6).

In other autoimmune diseases, mostly rheumatic diseases, such as juvenile idiopathic arthritis, systemic lupus erythematosus (SLE), and vasculitis, a relative high prevalence of primary antibody deficiencies has been described. More specifically, in a cohort of 72 children with SLE, 10 patients were retrospectively additionally diagnosed with either IgG2-, IgA-, or IgM-deficiency (7). Subsequent retrospective studies confirmed these previous results, with prevalence of primary antibody deficiencies ranging from 5.9 to 9.1% (8). In line, when investigating the prevalence of autoimmune diseases in patients with primary antibody deficiency, the prevalence of autoimmune disease is as high as 23%. This study also documented that in the majority of cases primary antibody deficiency preceded the onset of autoimmune disease (9). The development of autoimmune diseases in patients with antibody deficiency may be explained by the lack of IgG to trigger inhibitory signals through the FcγRIIB expressed by B cells. This hypothesis is supported by the observation of an enhanced autoantibody production in FcγRIIB-deficient mice in experimental models of arthritis (10, 11).

In contrast to rheumatic diseases, there are only few reports on total serum immunoglobulin concentrations in pemphigus and pemphigoid. So far, only one study investigated the total serum IgG and IgG1–4 concentrations in pemphigus vulgaris (PV) and pemphigus foliaceus (PF) (12), where a decreased total serum IgG was noted in both PV and PF patients. Regarding IgG subclasses, the relative abundance of IgG4 increased in both PV and PF, while a significant decrease in the relative IgG3 abundance was observed. Collectively, this study indicated that, like in rheumatic autoimmune diseases, a decrease in antibodies is also observed in pemphigus, which supports the notion that primary antibody deficiency may be associated with pemphigus. This data, however, was based on relatively small sample numbers and no data on serum immunoglobulin (Ig) concentrations in pemphigoid has been reported so far. In order to validate the reported data on IgG in pemphigus in a larger cohort, as well as to fill the knowledge gap regarding other Ig classes and pemphigoid diseases, we here contrasted the serum concentrations of total IgG, IgG1–4, IgA, and IgM in PV, PF, as well as the pemphigoid diseases bullous pemphigoid (BP) and linear IgA bullous dermatosis (LAD), to those in healthy controls.

## Methods

### Experiments With Human Biomaterials

Serum collections from patients and healthy volunteers were performed after written informed consent was obtained. Diagnosis of either disease was based on clinical presentation, direct immunofluorescent (IF) microscopy, and/or serological testing (4, 13, 14). All experiments with human samples were approved by the ethical committee of the Medical Faculty of the University of Lübeck and were performed in accordance with the Declaration of Helsinki.

### Patient and Control Cohorts

Demographics, results from direct IF microscopy, and serological findings are summarized in Supplement Table 1.

### Preanalytical Sample Processing

Approximately 9 mL of a donor's peripheral blood was collected by venipuncture in sterile tubes (S-Monovette®, Ref: 01.1601, Sarstedt, Nümbrecht, Germany), centrifuged for 15 min at 1,500× g at +4°C. The obtained serum was aliquoted, frozen, and stored at −20°C prior to use.

### Determination of Serum Ig Concentrations by ELISA

Serum concentrations of IgM, IgA, IgG, IgG1, IgG2, IgG3, or IgG4 were measured in sera of donors using commercially available ELISA kits (Thermo Fisher Scientific, Vienna, Austria, Cat. Nos. 88-50550-86, 88-50560-22, 88-50570-22, 88-50580-22, 88-50590-22, 88-50600-86, 88-50620-86, respectively) according to the manufacturer's recommendations with minor changes: At the preanalytical stage, a set of experiments on estimating precision, linearity of dilutions, and presence of high-dose hook effect were done. Intra-assay precision (r) was on average <10% in all analytical systems. To estimate inter-assay precision (Rw), different pooled sera of healthy donors of both genders (prepared in-house) were used as a positive control serum and added to each microtiter plate. Rw values for IgG, IgA, and IgM were estimated as 22.1, 17.8, and 18.7%, respectively. Additionally, in order to eliminate possible a high-dose hook effect and observe the analyte concentration within the analytical measurement range, 3–5 unknown sera and positive control serum were serially diluted for each analyte and linear ranges of dilutions for an Ig system were established (not shown) and 1:10,000, 1:10,000, and 1:1,000,000 dilutions were selected for IgM, IgA, and IgG, respectively. For IgG1, IgG2, IgG3, and IgG4 ELISA systems, 1:1,500, 1:200,000, 1:20,000, and 1:500 dilutions were used, respectively. Standards, positive control serum, and unknown serum samples were analyzed in duplicates. Absorbance was measured at λ = 450 nm with a microplate photometer (GloMax Discover System GM3000, Promega GmbH, Mannheim, Germany). A four-parameter logistic regression fitting curve was used to calculate concentration values (in milligrams per milliliter) after blank O.D. subtraction. The calculations were done with the help of MyAssays service (http://www.myassays.com/four-parameter-logistic-curve.assay). For technical reasons, adding the sum of IgG1–4 serum concentrations does not allow to determine the total IgG serum concentration (personal communication with Thermo Fisher).

### Data Availability

All raw data of this study and data and descriptive statistics of the demographic features of the study population are provided in Supplement Table 1.

### Statistical Analysis

**Figure 1:**
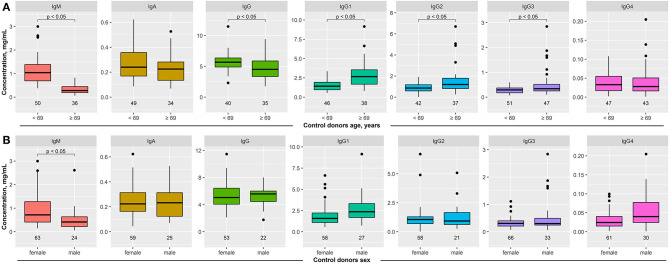
Impact of age and sex on serum immunoglobulin (Ig) concentrations in healthy blood donors. Serum concentration of IgM, IgA, IgG, and IgG1–4 was determined in healthy blood donors by ELISA. **(A)** The median age of the entire cohort was 69 years. To determine the impact of age on serum Ig concentration, these were compared between healthy subjects aged <69 years to those 69 years or older. In elderly healthy blood donors, IgM and IgG serum concentrations were decreased, while all immunoglobulin levels, with the exception of IgG4, were increased. **(B)** When stratified by sex, decreased IgM concentrations were noted in male healthy blood donors. All other serum Ig concentrations were similar between female and male healthy blood donors. Data are shown as median (black line) and 25/75 percentiles (boxes); the whiskers extend no further than 1.5× IQR from a hinge (where IQR is the interquartile range), and outliers (points). Welch test was used to test for statistically significant differences among the groups.

With the exception of one study (12), no data on Ig serum concentrations in pemphigus are available. Hence, we used this study to explore the variability of serum Ig concentrations. To allow statistical analysis, we aimed to include 100 samples per group. With the exception of BP (*n* = 99) and LAD (*n* = 55), this goal was reached for all groups. With R 3.6.2 (r-project.org) in the RStudio 1.2.5033 (rstudio.com) environment, we performed Welch's *t*-tests for age- (Figure 1A) and sex- (Figure 1B) related factors within the healthy (cntrl) group of donors. Having statistically significant differences in Ig concentrations established, subsequent 2- or 3-factor ANOVAs were performed with condition (5 levels: cntrl, PV, PF, BP, LAD) as a factor for IgG4; condition and age group (2 levels: younger than 69, 69 or older) as factors for IgA, IgG, IgG1, IgG2, and IgG3; and condition, age group, and sex (2 levels: female, male) as factors for IgM. For multiple pair-wise comparisons, Tukey's HSD *post-hoc* tests were performed. In all cases, two-tailed tests with statistical significance α = 0.05 were used.

## Results

### Age- and Sex-Differences in Ig Serum Concentrations

Sera from healthy donors were obtained from two different cohorts: One recruiting students at the University of Lübeck, as well as one existing cohort of elderly individuals (15). In order to control for either age or sex when comparing patient to control data, we used these cohorts to investigate the impact of age and sex on Ig serum concentrations. Regarding age, we stratified the entire cohort according to the median age (69 years). In controls, IgM and IgG serum concentrations were significantly decreased in the elderly (>69 years old), whereas IgG1–3 serum concentrations were increased. No difference in IgA and IgG4 concentrations was observed (Figure 1A). When stratified by sex, 69 healthy blood donors were females and 36 males. In contrast to age, sex had a much lesser impact on Ig serum concentrations. With the exception of IgM (lower concentration in males), no differences in Ig serum concentrations were noted (Figure 1B).

### Unaltered IgM Serum Concentrations in Patients With Pemphigus or Pemphigoid Disease

**Figure 2:**
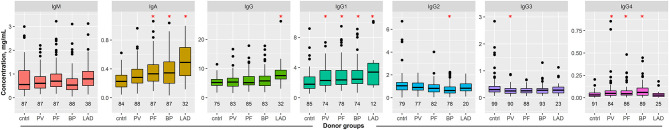
Serum immunoglobulin (Ig) concentrations in patients with pemphigus and pemphigoid disease. Serum concentration of IgM, IgA, IgG, and IgG1–4 were determined in healthy blood donors and patients with the indicated pemphigus and pemphigoid diseases by ELISA. The numbers below the boxes indicate the number of controls/patients included in each group. Data are shown as median (black line) and 25/75 percentiles (boxes); the whiskers extend no further than 1.5× IQR from a hinge (where IQR is the interquartile range) and outliers (points). ANOVA with Tukey's HSD procedure as *post-hoc* test was used for statistical analysis. *indicates *p* < 0.05 compared to controls.

In healthy controls, the median serum IgM concentration was 0.56 mg/ml. In all patient cohorts, the median IgM serum concentration was slightly higher. This difference, however, was not statistically significant (Figure 2, Supplement Tables 1, 2).

### Increase in Serum IgA Concentrations in Patients With Pemphigus Foliaceus, Bullous Pemphigoid, and Linear IgA Disease

In controls, interquartile serum IgA concentrations ranged from 0.15 to 0.31 mg/ml. In PF, BP, and LAD patients, a significant increase to 0.33, 0.34, or 0.49 mg/ml was observed, respectively (Figure 2, Supplement Tables 1, 2).

### Increased IgG Serum Concentration in Patients With Linear IgA Disease

Total IgG concentrations in all groups ranged from 5.19 to 5.77 mg/ml, with the exception of patients with linear IgA disease, where total serum IgG concentrations amounted to a median of 7.60 mg/ml (Figure 2, Supplement Tables 1, 2). When corrected for age and sex, this increase was significantly different to that of controls. Thus, the increase in total serum IgG concentrations in linear IgA patients seems to be associated with disease, rather than the lower age of this patient group.

### Increased IgG1 and IgG4 Serum Concentration in Patients With Pemphigus or Pemphigoid Disease

With the exception of IgG4 serum concentrations in LAD patients, a significant increase in serum IgG1 and IgG4 concentration was observed in all patient cohorts, when comparing to those observed in the controls (Figure 2, Supplement Tables 1, 2).

### Decreased IgG2 Serum Concentration in Patients With Bullous Pemphigoid

In BP patients, the median IgG2 serum concentration is 0.64 mg/ml, as opposed to controls, where the median IgG2 concentration is 1.05 mg/ml. For all other patient cohorts, IgG2 serum concentrations were similar to controls (Figure 2, Supplement Tables 1, 2).

### Decreased IgG3 Serum Concentration in Patients With Pemphigus Vulgaris

In PV patients, a slight but significant difference for total serum IgG3 concentrations was noted, specifically from a median of 0.30 mg/ml in controls to 0.25 mg/ml in PV (Figure 2, Supplement Table 2).

## Discussion

Based on the association of autoimmune diseases with primary antibody deficiencies and vice versa (7, 9), we had assumed to detect a high prevalence of antibody deficiency in pemphigus and pemphigoid diseases. Interestingly, we here document an increase in total serum IgG1 and IgG4 concentrations in almost all investigated patient cohorts, as well as an increase of IgA in PF, BP, and LAD patients. Interestingly, the increase in IgG4 serum concentrations may reflect a previous observation of an importance of Th2 responses in pemphigus (16). By contrast, IgG2 serum concentrations were significantly decreased in BP patients compared to controls, pointing toward a potential IgG2 deficiency in BP. More specifically, median IgG2 serum concentrations were reduced by almost 40% in BP patients. However, a prospective study would be required before implementing a screen for IgG2 deficiency in BP patients. Assuming a 35% decrease in IgG2 serum concentrations in BP compared to age- and sex-matched controls, a similar variability as observed herein (90%), and aiming for a high power of 90% and α = 0.05, IgG2 serum concentrations need to be analyzed in 140 BP patients and 140 controls. In our cohort, the IgG2 deficiency manifested despite normal serum concentrations of total IgG. However, the concentrations of IgG1 and IgG4 were increased, which may have balanced the total IgG serum concentrations in BP patients. This increase of total IgG1 and IgG4 may reflect the autoantibody production, as IgG1 and IgG4 are the major IgG subclasses in BP (17). IgG2 subclass deficiency puts the affected people at a high risk for recurrent infections, especially the respiratory tract (18). Given that infections are common adverse events in BP patients, as well as one major cause of death (19, 20), which have so far been attributed to the corticosteroid treatment, detection of IgG2 deficiency, with subsequent substitution therapy, may improve the overall outcome of BP. Furthermore, decreased IgG3 serum concentrations were noted in PV patients. However, this decrease was not as prominent as the reduced IgG2 concentrations in BP, but this finding also warrants follow-up and validation in prospective studies. Of note, patients with primary complement deficiencies are also prone to developing autoimmune diseases (21, 22). In BP patients, plasma concentrations of C3a, C4a, and C5a are identical to those of age- and sex-matched controls (23). Thus, a primary complement deficiency seems unlikely in BP, but current data does not allow to fully exclude complement deficiencies in BP.

The observed increase in IgA serum concentration in BP, PF, and LAD as well as the increase of IgG1 and IgG4 in almost all patient cohorts is most likely due to the autoantibody production, as IgA is the predominant Ig in LAD (24), and IgG1 and IgG4 are among the predominant IgG subclasses in PV, PF, and BP (25–27). In line with this assumption, Dsg3 autoantibodies have been shown to contribute as much as 5% of the total IgG4 serum concentration (12). Our findings, however, are partially in contrast to those reported previously for pemphigus, where in both PV and PF, significantly lower total IgG serum concentrations are reported (12). In this previous study, however, the reported serum IgG concentration amounted to 28 mg/ml, which is relatively high compared to the reported range of 7–16 mg/ml in healthy volunteers (28, 29), whereas the healthy blood donors in our study had IgG values at the lower end of the reported range. Independent of the method used to detect serum Ig, within a given set of experiments, this would (if at all) be a systematic error affecting all groups. Thus, differences should still be detectable, if the measurements are within the detection limit of the used test.

This study is not without limitations. Foremost, the retrospective and single center design may have led to confounding of the data. In addition, the patient and control groups were not matched for age and sex, which was, however, accounted for by considering age or sex when deciding to use 1-, 2-, or 3-way ANOVA. In light of different data on IgG concentrations in pemphigus presented here and by Funakoshi et al. (12), this must be considered when interpreting the data. Furthermore, as no data on antibody concentrations have been published, thus, in our opinion, both studies have to be considered as exploratory. However, with the exception of one study with a sole focus on pemphigus (12), no data has been available regarding serum Ig concentrations in pemphigus and pemphigoid. Hence, despite these shortcomings, the results obtained provide the basis allowing to plan prospective diagnostic trials. Among the detected differences, the almost 40% reduction of IgG2 in BP patients should be evaluated in a prospective multicenter clinical trial because appropriate detection and management of IgG2 deficiency in BP patients would have major clinical implications and have the potential to improve the overall patient outcome.

## Data Availability Statement

All datasets generated for this study are included in the article/Supplementary Material.

## Ethics Statement

The studies involving human participants were reviewed and approved by Ethical Committee of the Medical Faculty of the University of Lübeck, and were performed in accordance with the Declaration of Helsinki. The patients/participants provided their written informed consent to participate in this study.

## Author Contributions

SK: performed experiments. KB, NB, AV, DZ, and ES: obtained and characterized patient and control serum. SK and RL: statistical analysis. RL: conception of manuscript. All authors: writing and revising the manuscript.

## Conflict of Interest

RL, DZ, and ES have received research funding and fees for consulting or lecturing from Biotest AG. The remaining authors declare that the research was conducted in the absence of any commercial or financial relationships that could be construed as a potential conflict of interest.
