# Supplementary material for: Alterations of Total Serum Immunoglobulin Concentrations in Pemphigus and Pemphigoid: Selected IgG2 Deficiency in Bullous Pemphigoid
Source: Front Med (Lausanne). 2020 Sep 2;7:472. doi: 10.3389/fmed.2020.00472 (PMC7493804; doi:10.3389/fmed.2020.00472)
Supplement: Supplementary file 2 [file Table_2.docx]

|  | **IgM (mg/ml)** | **IgA (mg/ml)** | **IgG (mg/ml)** | **IgG1 (mg/ml)** | **IgG2 (mg/ml)** | **IgG3 (mg/ml)** | **IgG4 (mg/ml)** |
| --- | --- | --- | --- | --- | --- | --- | --- |
| **Control** | 0.56 (0.35; 1.14) | 0.23 (0.15; 0.31) | 5.22 (4.22; 6.30) | 1.79 (1.23; 2.77) | 1.05 (0.69; 1.34) | 0.30 (0.21; 0.42) | 0.03 (0.02; 0.05) |
| **PV** | 0.61 (0.41; 0.89) | 0.28 (0.20; 0.40) | 5.39 (3.95; 6.73) | **2.29 (1.65; 3.62)** | 0.91 (0.67; 1.29) | **0.25 (0.16; 0.35)** | **0.05 (0.03; 0.08)** |
| **PF** | 0.70 (0.47; 1.00) | **0.33 (0.23; 0.46)** | 5.19 (4.38; 7.29) | **2.36 (1.74; 3.63)** | 0.82 (0.51; 1.14) | 0.26 (0.18; 0.34) | **0.05 (0.03; 0.08)** |
| **BP** | 0.53 (0.34; 0.80) | **0.34 (0.20; 0.50)** | 5.77 (4.13; 7.40) | **2.46 (1.87; 3.63)** | **0.64 (0.41; 0.95)** | 0.28 (0.17; 0.34) | **0.06 (0.03; 0.11)** |
| **LAD** | 0.79 (0.51; 1.11) | **0.49 (0.31; 0.70)** | **7.60 (6.42; 9.44)** | **3.40 (1.70; 4.57)** | 0.84 (0.60; 1.22) | 0.28 (0.18; 0.40) | 0.03 (0.01; 0.05) |

**Supplement Table 2.** Serum immunoglobulin (Ig) concentrations in patients with pemphigus and pemphigoid disease. Serum Ig concentrations were analyzed by ELISA in patients with pemphigus vulgaris (PV), pemphigus foliaceus (PF), bullous pemphigoid (BP), linear IgA disease (LAD), as well as healthy controls. Numbers in the table correspond to the median (25%; 75% percentile) mg/ml Ig concentration. Characteristics of the cohorts are described in supplement table 1; statistical analysis was performed using 1-, 2-, or 3-Way ANOVA, depending on the impact of age and sex factors on Ig concentrations (Fig. 1a-b) with Tukey’s HSD procedure as *post hoc* tests. Compared to controls, we noted an increased serum IgA concentration in LAD, an increase of IgG1 in all patient cohorts, a decrease in IgG2 in BP patients, and an increase of IgG4 in PV, PF and BP. Significant differences (in relation to controls) are indicated by bold font and light gray shading. Total IgM, IgG and IgG3 serum concentrations were not significantly different in all patient samples compared to controls.
